# Applied techniques for putting pre-visit planning in clinical practice to empower patient-centered care in the pandemic era: a systematic review and framework suggestion

**DOI:** 10.1186/s12913-021-06456-7

**Published:** 2021-05-13

**Authors:** Marsa Gholamzadeh, Hamidreza Abtahi, Marjan Ghazisaeeidi

**Affiliations:** 1 Health Information Management Department, School of Allied Medical Sciences, Tehran University of Medical Sciences, 5th Floor, Fardanesh Alley, Qods Ave, Tehran, Iran; 2 Pulmonary and Critical care Medicine Department, Thoracic Research Center, Imam Khomeini Hospital, Tehran University of Medical Sciences, Tehran, Iran

**Keywords:** Pre-visit, Patient-centered care, Patient care planning, Framework

## Abstract

**Background:**

One of the main elements of patient-centered care is an enhancement of patient preparedness. Thus, pre-visit planning assessment tools was emerged to prepare and involve patients in their treatment process.

**Objective:**

The main objective of this article was to review the applied tools and techniques for consideration of putting pre-visit planning into practice.

**Methods:**

Web of Science, Scopus, IEEE, and PubMed databases were searched using keywords from January 2001 to November 2020. The review was completed according to the Preferred Reporting Items for Systematic Reviews and Meta-Analyses checklist. Then, qualitative analysis was done to suggest an appropriate framework by mapping the main concepts.

**Results:**

Out of 385 citations were retrieved in initial database searches, 49 studies from ten countries were included. Applied pre-visit techniques can be classified into eight categories. Our results showed that almost 81% of studies were related to procedures that were done between each visit, while 42% of articles were related to before visits. Accordingly, the main approach of included articles was patient preparedness. While 38 studies reported this approach is effective, three studies reported the effectiveness of such tools as moderate, only two articles believed it had a low effect on improving patient-centered care.

**Conclusion:**

This survey summarized the characteristics of published studies on pre-visit planning in the proposed framework. This approach could enhance the quality of patient care alongside enhancement patient-provider communication. However, such an approach can also be helpful to control pandemic diseases by reducing unnecessary referrals.

**Supplementary Information:**

The online version contains supplementary material available at 10.1186/s12913-021-06456-7.

## Background

In the information-driven care era, although the ultimate goal of health systems is still improving the quality of patient care, the patient care model has shifted from personal responsibility to participatory medical decision-making [1]. Thus, the responsibility of the patient’s health is no longer solely with the physician. On the other hand, the role of the patient in promoting his health status cannot be denied [2]. Hence, the patient-centered care (PCC) model was introduced to show the participatory role of the patient and other health care providers in the process of treatment and patient care [3–6]. Since the PCC idea was introduced, various definitions and models have been proposed to distinguish the main elements of this model [5, 7–12]. Up to now, the best model that has been able to explain the main components of such a care model is the model presented by the Picker Institute [13]. This model consists of eight parts that outline the factors affecting the achievement of an optimal patient-centered care model [5, 12, 14].

One of the main elements of the PCC approach is respect for patients’ value by preparation of patients for each visit [6]. Sometimes patients have to spend more time in the waiting room than in a physician’s office [15, 16]. Also, in each appointment, especially in the first visit, more than 5 min should be devoted to determining who the patient is, what is his problems, which drugs she/he used, what is his/her medical history, and so on [17]. This process is so complex in patients who have a chronic condition or patients with multiple chronic conditions with multiple medications [18, 19]. It can be useful to prevent the spread of the disease. Limited time for each visit and patient complexity might have a negative impact on the patient-physician relationship.

In this context, pre-visit planning and visit preparation concepts have been suggested by American Medical Association (AMA) as a solution to address these challenges. It can help physicians when the patient checks in for the first time, he is already behind [20]. This term (pre-visit planning) was introduced by Sinsky et al. in 2014 to collect and organize patient data before a patient visit [21].

**Fig. 1:**
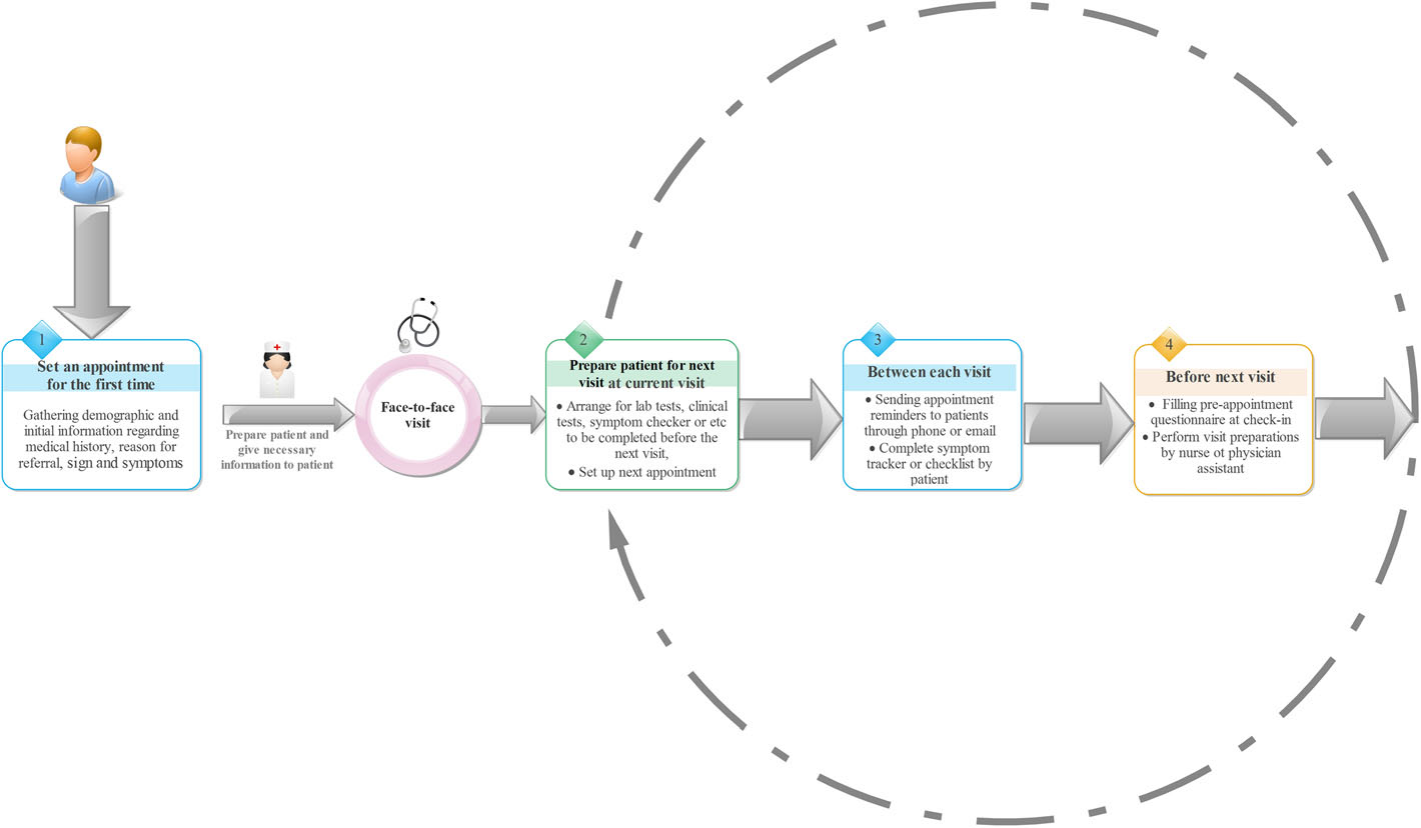
The conceptual model of pre-visit planning

The purpose of pre-visit planning is to help the patient and physician to save time and improve care by gathering and organizing information in a structured way. Therefore, a health care provider can pay more attention to interpretation, discussion, and response to a patient during the visit. This idea is not just to plan ahead before each visit. Dr. Sinsky explains that pre-visit planning could include a broader concept that could generally refer to preparing the patient for a face-to-face visit more effectively [21]. The pre-visit planning concept is described in Fig. 1 as a conceptual model.

However, there are various methods to apply this new approach into practice, it usually includes scheduling future appointments and preparing patients before the visit [22]. These techniques are known as pre-visit assessment tools. The use of pre-visit assessment tools focuses on involving the patient and the physician through the patient care process [23]. As it is apparent in Fig. 1, it can occur at end of each visit, arranging for the next visit, programming for the next clinical and paraclinical testing, gathering the necessary information for the subsequent visits, and take steps regarding the handoff of patients [24].

With pre-visit planning, patients and physicians are prepared to make meaningful use of their time during each visit. Furthermore, patients could have an impressive role in clinical decision-making regarding their treatment process [25]. Hence, several studies have focused on the power of patient-centered care to improve patient care, but no studies have been published to examine the applying pre-visit planning techniques in the context of patient-centered care. The main objective of this study is to review the consideration of pre-visit planning used in patient-centered care. Throughout this paper, the term pre-visit planning will refer to any intervention, care program, patient-centered planning, or even educational plan that is considered to prepare the patient for a face-to-face visit or improve the patient-provider relationship. Specific aims of this survey are as follows: 1) representing an overview of applied methods regarding pre-visit planning with their characteristics in published studies, 2) to investigate the published studies on applying pre-visit planning regarding clinical aspects such as type of disease, 3) to determine the effectiveness of putting pre-visit planning into routine practice, 3) providing an overview of the sample size, approaches, and collected information concerning applied methods and techniques, 4) suggesting a framework in this context.

## Method

A systematic search of four databases (Web of Science, Scopus, IEEE, and PubMed) was conducted from January 2001 to November 2020 using keywords alongside Mesh terms. These databases were selected for their inclusion of qualitative studies and health research. The keywords used in the search strategy were drawn from preliminary searches according to our study goals. Those keywords were validated and additional keywords added by checking the terms used in articles identified in preliminary searches. Boolean search strategies were described in Additional file 1: Table A-1. Since no result was found in the IEEE database, it was removed from source databases in Table A-1. This systematic review was completed according to the Preferred Reporting Items for Systematic Reviews and Meta-Analyses (PRISMA) checklist to ensure the inclusion of relevant studies [26].

### Inclusion and exclusion criteria for study selection

Articles were included if they met the following criteria: 1) The focus of the study was on applying the pre-visit approach through the patient care process. 2) Population includes all of the patients with any type of disease, 3) This study covered all phases of the patient care process, 4) Published in recent 10 years and matched with the search query, 5) Limited to those published in the English language, 6) Only published articles and reviews in peer-reviewed journals were included, 7) All type of study designs, 8) Improve patient-centered care, 9) Studies that received an acceptable score in terms of quality based on the checklist. Articles excluded if they met the following criteria: 1) The title, abstract, or full text of the article did not relate to pre-visit planning, 2) Thesis, book chapters, letters to editors, short briefs, reports, technical reports, book reviews, review, or meta-analysis, 3) Non-English papers, 4) Publication that their full-text is not available.

### Data screening phase

Based on our search strategy; articles were retrieved from databases. Additionally, related studies were added manually by a simple search in Google Scholar and reference checking. All of the citations were imported to EndNote software for better resource management. Then, duplicated articles were removed. In the first phase, all titles and abstracts of articles were examined based on our main objective to select relevant studies by one author (MG). A second reviewer (MGH) reviewed a sample of studies randomly. After that, the full texts of relevant studies were screened thoroughly by two reviewers (MG and MGH). If there was a disagreement between the authors in the selection of relevant studies, the final decision was made by HA. Lastly, some studies remained as eligible articles for qualitative analysis. The extraction forms were designed by researchers to manage and investigate the obtained information. To diminish bias, key subjects of articles summarized and entered into customized extraction forms based on specific classifications. Two authors (MG and MGH) independently extracted the study characteristics based on the classification. The information extracted by the researchers was re-examined to reach an agreement. The next reviewer (HA) assessed and verified the extracted information.

### Critical quality appraisal

The methodological quality of the included articles was evaluated using the Qualitative research Critical Appraisal Program (CASP) tool by two authors. This instrument was used in systematic reviews frequently for qualitative synthesis [27]. It was employed for appraising the strengths and limitations of any qualitative research methodology. It was recommended for health-related researches and it is appropriate for novice researchers [28]. Critical appraisal was performed independently by two researchers.

### Analysis

To extract some necessary information, specific categories were considered to classify and analyze relevant articles. All of the articles were synthesized regarding general and specific domains. The general domain comprises the title, author, year of publication, journal name, type of study, the main objectives. Accordingly, the specific domain comprises applied pre-visit techniques, disease, clinic, sample size, country, outcome measures, effectiveness, and collected data. Analysis of the extracted information from eligible articles and framework suggestions were conducted based on these predefined categories.

## Results

**Fig. 2:**
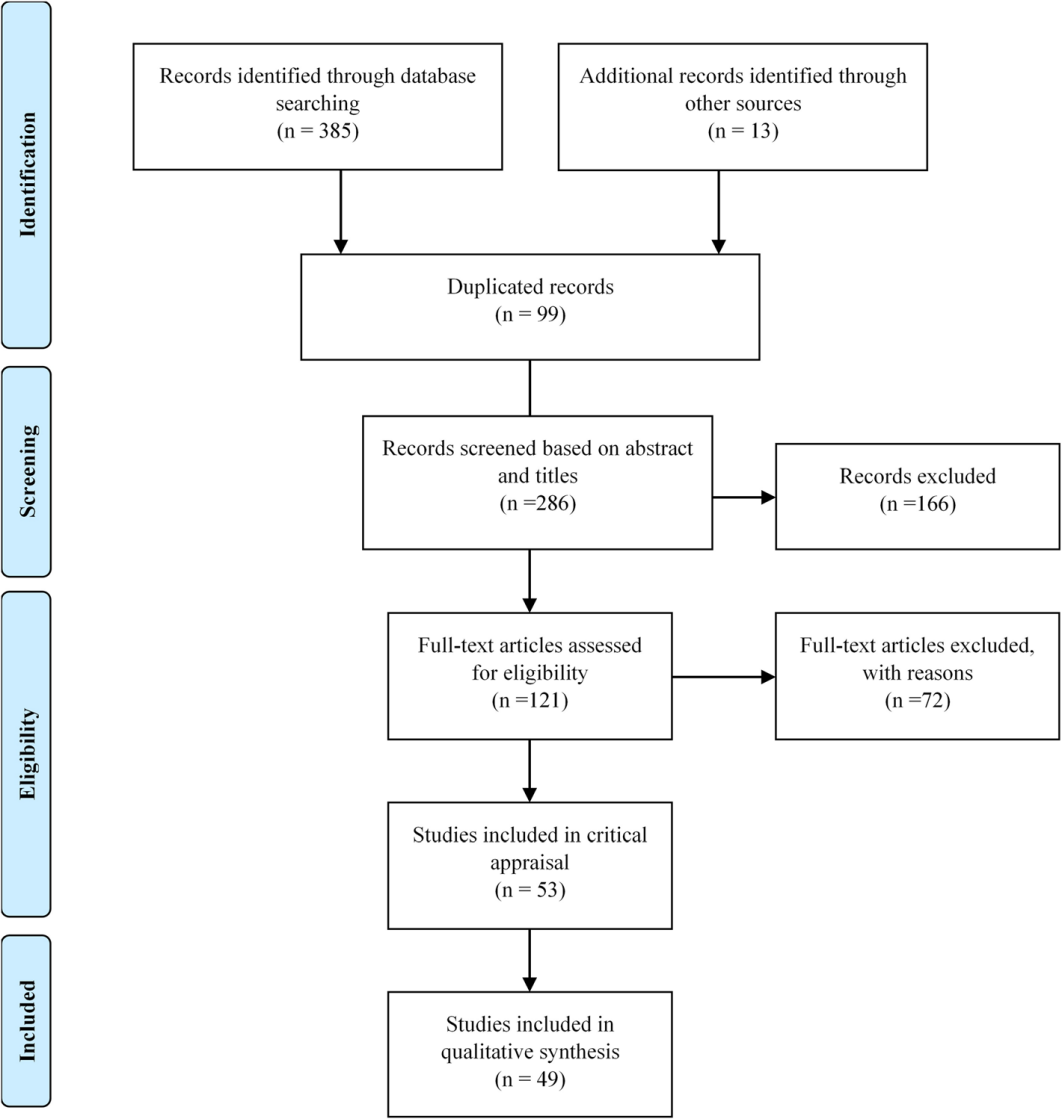
PRISMA workflow for summarizing the selection of papers process

In total, systematic literature searching of databases yielded 385 citations. Of which 99 articles were removed due to duplication. Next, one hundred and sixty-six papers were excluded after screening titles and abstracts. In the following, 72 papers were excluded after full-text reading. Finally, 49 papers are identified as an eligible article which met our inclusion criteria. The screening process for articles based on the PRISMA checklist is shown in Fig. 2. All included papers had the minimum score (10 from 20) of quality assessment using the CASP tool. Only four papers were excluded based on quality appraisal assessment. Therefore, forty-nine articles were identified as eligible studies for qualitative analysis.

### General characteristics

All included studies are published in journals from 2001 to 2020. The trend of publishing articles in this field was following an upward trend. In terms of the type of study, studies were conducted in different designs. Most of which were clinical trial studies. The descriptive analysis regarding the type of study in the included articles is represented in Table 1. In the following, the results of the review of studies by author, year of publication, the main objectives, the sample size, type of pre-visit planning, clinic, the effectiveness of the applied method, and outcome of using the pre-visit planning are summarized in Table 2.

**Table 1:** The frequency of different types of study

Study type	Frequency	Percentage	References
RCT	21	44.9%	[22, 23, 29–47]
Before-after study	10	18.4%	[48–57]
Descriptive study	9	18.4%	[25, 58–65]
Cross-sectional study	2	4.1%	[66, 67]
Mixed method	2	4.1%	[68, 69]
Cohort	1	2.0%	[24]
Non-Randomized trials	1	2.0%	[70]
Quasi-experimental study	1	2.0%	[71]
Sequential prospective study	1	2.0%	[72]
Time-series analysis	1	2.0%	[73]

**Table 2:** Summary of reviewed articles and evidence

#	Author	Year	Journal	Pre-visit model	Objective	Findings and applied techniques characteristics								CASP SCORE
						Sample size	Effectiveness	Clinic	Country	Disease	Type of medical informatics solution	Collected data	Outcome measures	
1	Allende-Richter, S. H. et al. [64]	2018	*Clin Pediatr*	*Paper-based checklist*	(1) Enhance team working among care team members and (2) Provide early access to existing medical services.	Not mentioned	+++	Primary care clinic	USA	General	Pre-assessment tools	Demographic data, Medical history, Family history, Reason for referral, Symptoms, Medication	Patient-provider communication, Perceived involvement in care, Patient satisfaction, Identifying referral appropriateness	18
2	Rivo, J. et al. [24]	2015	*Popul Health Manag*	*Phone-based pre-office visit preparation*	Improving compliance with recommended tests and screenings.	7491 patients	+++	Primary care clinic	USA	Diabetes	Decision aid tools	Demographic data, Reason for referral, Symptoms	Patient-provider communication, Perceived involvement in care, Patient expectations in consultations, Adherence to visit scheduling	17
3	Cox, N et al. [57]	2018	*J Am Board Fam Med*	*A pre-clinic care team consultation*	To evaluate the impact of a pre-visit pharmacist consultation for chronic non-cancer pain	45 patients	+++	A family medicine residency clinic	USA	Chronic Opioid	Pre-assessment tools	Demographic data, Reason for referral, Symptoms	Patient satisfaction, Patient expectations in consultations, Appointment intake information, Medication and treatment adherence, ITT analysis, Mental health topics	16
4	Paget et al. [53]	2015	*Health Promot Pract,*	*Phone-based pre-office visit preparation*	To increase patient compliance with scheduled appointments, follow up, and complete exams on time.	5539 patients	+++	Diabetic clinic	USA	Diabetes	Decision aid tools	Demographic data, Reason for referral, Symptoms	Illness perceptions, Perceived involvement in care, Patient satisfaction, Patient expectations in consultations, Medication and treatment adherence, Adherence to visit scheduling, Visit length	17
5	Bose-Brill, S et al. [70]	2018	*J Med Internet Res*	*EHR-linked care program*	To determine the impact of pre-visit ACP planning using a secure EHR-linked framework	419 patients aged between 50 and 93 years	+++	Routine follow-up visit	USA	Primary care clinic	Decision aid tools, Pre-assessment tools, Reminders	Demographic data, Medical history, Lifestyle, Family history, Lab data, Reason for referral, Patient awareness, Drug side effects Symptoms, Medication	Patient-provider communication, Illness perceptions, and knowledge, Patient expectations in consultations, Medication and treatment adherence, visit length, Identifying referral appropriateness	15
6	Riese, A et al. [48]	2015	*Acad Pediatr*	*Electronic pre-office visit checklist*	To determine the efficacy of electronic pre-visit questionnaires (PVCs)	183 adolescents	+++	Pediatric primary care clinic	USA	pediatric diseases	Pre-assessment tools	Demographic data, Medical history, Family history, Reason for referral, Symptoms, Medication	Illness perceptions, Perceived involvement in care, Patient satisfaction, Identifying referral appropriateness	18
7	Myers, P et al. [36]	2020	*J Plast Reconstr Aesthet Surg*	*Online and offline sources of information and support*	To improve patient understanding of insurance coverage by providing educational materials	100 patients	++	Surgery clinic	USA	Obesity	Patient education	Demographic data, Reason for referral, Patient awareness, Drug side effects Medication	Patient satisfaction, Appointment intake information, Quality of life	16
8	Frank, O et al. [50]	2014	*Aust Fam Physician*	*Paper-based checklist*	To assess whether ongoing programs are acceptable to patients and feasible in busy routine clinical practice.	14 GP and 130 patients	+++	General clinic	Australian	General	Pre-assessment tools	Demographic data, Medical history, Family history, Reason for referral, Symptoms, Medication	Patient-provider communication, Patient satisfaction, Patient expectations in consultations, Identifying referral appropriateness	13
9	Lewin, W et al. [51]	2009	Can Fam Physician	*Paper-based checklist*	To assess the efficacy of a pre-visit questionnaire (PVQ)	210 patients aged 13 to 19	+++	Primary care	Canada	Psychology	Pre-assessment tools	Demographic data, Medical history, Family history, Reason for referral, Symptoms, Medication	Patient-provider communication, Illness perceptions, Patient satisfaction, Patient expectations in consultations, Identifying referral appropriateness	18
10	Liu, T et al. [25]	2018	J Arthroplasty	*Electronic pre-office visit checklist*	To clarify the patient preference with hip and knee arthritis regarding pre-visit completion	51 Patients	++	Arthroplasty clinics	USA	Hip and Knee Pain	Pre-assessment tools, Decision aid tools	Demographic data, Medical history, Lifestyle, Family history, Lab data, Reason for referral, Symptoms, Medication	Illness perceptions and knowledge, Perceived involvement in care, Patient waiting times, Identifying referral appropriateness	18
11	Stankowski-Drengler, T. J et al. [37]	2019	Ann Surg Oncol	*Online and offline sources of information and support*	To assess completion, delivery method, and barriers or facilitators to pre-visit completion	201 patients	++	Cancer clinic	USA	Breast cancer	Patient education	Demographic data, Reason for referral, Patient awareness, Symptoms	Illness perceptions, Perceived involvement in care, Appointment intake information	14
12	Wald, J. S et al. [47]	2010	J Am Med Inform Assoc	*EHR-linked pre-visit checklist*	To examine the impact of pre-visit electronic journals in primary care as a decision aid	2027 patients and 272 physicians	+++	Primary clinic	USA	General	Decision aid tools, Pre-assessment tools, Reminders	Demographic data, Medical history, Lifestyle, Family history, Lab data, Reason for referral, Patient awareness, Drug side effects Symptoms, Medication	Patient-provider communication, Illness perceptions, Perceived involvement in care, Patient satisfaction, Patient expectations in consultations, Identifying referral appropriateness	20
13	Zanini, C et al. [65]	2018	Patient Educ Couns	*Paper-based checklist*	Assess high-quality websites on patients’ perceptions of	38 patients	+++	neurology	Switzerland	Chronic pain	Pre-assessment tools	Demographic data, Medical history, Family history, Lab data, Reason for referral, Symptoms	Patient-provider communication, Patient expectations in consultations	15
14	Grant. R et al. [22]	2016	Contemp Clin Trials	*EHR-linked care program*	To determine a strategy for improving diabetes care	146 physicians with 2496 of their patients	+++	Primary care clinic	Canada	Diabetes	Decision aid tools, Pre-assessment tools, Reminders	Demographic data, Medical history, Lifestyle, Family history, Lab data, Reason for referral, Patient awareness, Drug side effects Symptoms, Medication	Illness perceptions, Perceived involvement in care, Medication and treatment adherence, Medication and treatment adherence, Symptom control	19
15	Frank, O. R et al. [56]	2011	BMC Fam Pract	*Automatic reminders and sheets*	Assess satisfaction with the decision process	Sixty patients	+++	Primary care clinic	Australia	General	Reminders	Demographic data, Medication	Patient satisfaction, Medication and treatment adherence, Adherence to visit scheduling	18
16	Rodenbach, R et al. [38]	2017	J Clin Oncol	*Online and offline sources of information and support*	To examine the impact of a decision aid versus high-quality websites	24 oncologists and 170 patients	+++	Oncology clinic	USA	Cancer	Decision aid, Patient education	Demographic data, Drug side effects	Illness perceptions, Patient satisfaction, Patient expectations in consultations, Appointment intake information	17
17	Hitchings, S., and Barter, J [54].	2009	J Fam Plann Reprod Health Care	*Self-triage or self-assessment tool*	This study examined whether and how a pre-consultation sheet (PCS) can facilitate doctors in identifying targets for medical advice.	193 patients	+++	sexual health clinics	UK	Sexual problems	Pre-assessment tools	Demographic data, Medical history, Lifestyle, Family history, Lab data, Reason for referral, Patient awareness, Drug side effects Symptoms, Medication	Patient-provider communication, Illness perceptions, Perceived involvement in care, Adherence to visit scheduling, ITT analysis, Self-care, Mental health topics, Identifying referral appropriateness Symptom control	19
18	Sleath, B et al. [23]	2017	Patient Educ Couns	*Online and offline sources of information and support*	To improve patient-provider communication during time-limited primary care visits and represent a strategy for improving diabetes care.	259	+++	pediatric asthma clinic	USA	Asthma	Patient education	Demographic data, Patient awareness	Patient-provider communication, Appointment intake information	15
19	Tucholka, J. L. et al. [39]	2018	J Am Coll Surg	*Online and offline sources of information and support*	To assess the acceptability of a new strategy of pre-consultation prevention summaries and reminders in general practice.	377 patients	+++	Breast cancer clinic	USA	Breast cancer	Patient education	Demographic data, Patient awareness	Illness perceptions, Medication and treatment adherence	16
20	Aboumatar, H. J et al. [66]	2013	J Gen Intern Med	*Online and offline sources of information and support*	Combining patient-oncologist intervention to improve communication in advanced cancer	41 primary care physicians and 275 of their patients	+++	Primary care clinic	USA	Hypertension	Patient education	Demographic data, Patient awareness	Appointment intake information	18
21	Albada, A. et al. [29]	2015	Patient Educ Couns	*Electronic pre-office visit checklist*	To help reduce waiting times and duplication of work, improve patient pathways and decrease wasted visits	197 patients	+	Breast cancer clinic	Norway	Breast cancer	Decision aid tools, Pre-assessment tools, Reminders	Demographic data, Medical history, Family history, Reason for referral, Symptoms, Medication	Patient-provider communication, Perceived involvement in care, Adherence to visit scheduling, Visit length	18
22	Bruce, J. et al. [60]	2018	J Cancer Educ	*Online and offline sources of information and support*	To evaluate teen feedback on an asthma question intervention designed to motivate teens to be more engaged during visits and	377 patients	++	breast surgery clinic	USA	Breast cancer	Patient education	Patient awareness, Symptoms	Appointment intake information, Identifying referral appropriateness	15
23	Walker, M. E. et al. [52]	2018	J Hand Surg Asian Pac	*Paper-based checklist*	To compare patients’ knowledge after the pre-consultation delivery of standard websites versus a web-based decision aid (DA).	71 patients	++	Surgery clinic	USA	Hand problems	Pre-assessment tools	Demographic data, Medical history, Reason for referral, Symptoms	Patient-provider communication	14
24	Savage, C. et al. [69]	2019	Int J Qual Health Care	*Paper-based checklist*	To elucidate how HL influences patients’ interest in participating in medical visit communication.	289 questionnaires	+++	Primary clinic	Sweden	General	Pre-assessment tools	Demographic data, Medical history, Reason for referral, Symptoms	Patient-provider communication, Perceived involvement in care	14
25	Judson, T. J. et al. [55]	2020	J Am Med Inform Assoc	*Self-triage or self-assessment tool*	To prepare for breast cancer genetic counseling.	950 unique patients	+++	the large academic health system	Canada	COVID-19	Pre-assessment tools	Demographic data, Medical history, Lifestyle, Family history, Lab data, Reason for referral, Drug side effects Symptoms, Medication	Perceived involvement in care, Self-care, Self-care, Symptom control	18
26	Albada, A. et al. [30]	2012	Genet Med	*Electronic pre-office visit checklist*	To test an approach for delivering web-based information to breast cancer patients.	200 counselees	+++	Breast cancer genetic counseling clinic	Netherlands	Breast cancer	Decision aid tools, Pre-assessment tools	Demographic data, Medical history, Lifestyle, Family history, Lab data, Reason for referral, Drug side effects Symptoms, Medication	Patient-provider communication, Appointment intake information, Adherence to visit scheduling	18
27	Purkaple, B. A et al. [45]	2016	Ann Fam Med	*Paper-based checklist*	To measure hand surgery patient understanding compared with a US academic hand surgery practice	64 encounters	++	Primary clinic	USA	General	Pre-assessment tools	Demographic data, Medical history, Lifestyle, Family history, Lab data, Reason for referral, Symptoms, Medication	Patient-provider communication	13
28	Krist, A. H. et al. [40]	2007	Ann Fam Med	*Online and offline sources of information and support*	To explore how the See-and-Treat concept can be applied in primary care and its effect	497 participants	++	Primary clinic	USA	Prostate cancer	Patient education	Demographic data, Patient awareness	Patient expectations in consultations, visit length, Identifying referral appropriateness	17
29	Fothergill, K. E. et al. [68]	2013	Acad Pediatr	*Electronic pre-office visit checklist*	To direct patients to targeted intake, advice, information, and care for respiratory symptoms and COVID-19 concerns	172 parents	++	primary care pediatric	USA	Mental health	Decision aid tools, Pre-assessment tools, Patient education	Demographic data, Medical history, Lifestyle, Family history, Lab data, Reason for referral, Patient awareness, Drug side effects Symptoms, Medication	Patient-provider communication, Illness perceptions, Appointment intake information, Patient waiting times, Mental health topics	11
30	Lee, Y. K et al. [58]	2017	J Eval Clin Pract	*Electronic pre-office visit checklist*	To address the unmet needs of patients with chronic diseases regarding the pre-visit website	15 participants	+++	Primary care	Malaysia	Chronic disease	Decision aid tools, Pre-assessment tools, Patient education	Demographic data, Medical history, Lifestyle, Family history, Lab data, Reason for referral, Drug side effects Symptoms, Medication	Illness perceptions, Perceived involvement in care, Appointment intake information, Identifying referral appropriateness	20
31	Johansen, M. A. et al. [59]	2011	Methods Inf Med	*Electronic pre-office visit checklist*	To wonder if patients could encourage primary care physicians by writing goals on pre-encounter forms.	83 respondents	+++	visiting university locations	Norway	General	Decision aid tools, Pre-assessment tools, Patient education	Demographic data, Medical history, Lifestyle, Family history, Lab data, Reason for referral, Drug side effects Symptoms, Medication	Patient-provider communication, Illness perceptions, Patient expectations in consultations	11
32	Hu, X et al. [61]	2012	J Health Commun	*Online and offline sources of information and support*	To evaluate whether pre-visit educational decision aids facilitate shared decision making.	505 respondents	+++	primary care	USA	General	Patient education	Demographic data, Patient awareness	Patient expectations in consultations, Appointment intake information	15
33	Albada, A. et al. [42]	2012	Fam Cancer	*Online and offline sources of information and support*	To evaluate how parents and physicians perceive the utility of a comprehensive, electronic pre-visit screening, and its impact on the visit.	371 counselees	+++	Breast cancer genetic counseling clinic	Netherlands	Breast cancer	Patient education	Demographic data, Patient awareness	Patient satisfaction, Medication and treatment adherence, Illness perceptions, and knowledge	15
34	Frost, J. et al. [31]	2019	BMJ Open	*Electronic pre-office visit checklist*	To explore the impact of a pre-consultation website in addressing patients’ unmet needs during chronic disease consultations.	120 patients and 15 diabetologists	+++	Diabetes clinics	UK	Diabetes	Decision aid tools, Pre-assessment tools, Patient education	Demographic data, Medical history, Lifestyle, Family history, Lab data, Reason for referral, Drug side effects Symptoms, Medication	Appointment intake information, Visit length	17
35	O’Brien, M et al. [32]	2017	BMC Fam Pract	*Electronic pre-office visit checklist*	To investigate people’s attitude towards providing symptom information electronically before a consultation.	831 patients	+	Family physician’s clinic	Canada	Lung cancer	Decision aid tools, Pre-assessment tools, Patient education	Demographic data, Medical history, Lifestyle, Family history, Lab data, Reason for referral, Drug side effects Symptoms, Medication	Illness perceptions, Patient expectations in consultations, Appointment intake information, Medication and treatment adherence, Mental health topics	20
36	Wald, J. S. et al. [43]	2009	AMIA Annu Symp Proc	*EHR-linked care program*	To investigate the potential of e-journal to improve patient care during a visit	126 patients and 230 primary care providers	+++	Primary care	USA	Diabetes	Decision aid tools, Pre-assessment tools, Patient education, Reminders	Demographic data, Medical history, Lifestyle, Family history, Lab data, Reason for referral, Drug side effects Symptoms, Medication	Appointment intake information, Self-care, Symptom control, Visit length,	16
37	Albertson, G. et al. [72]	2002	Am J Manag Care	*Paper-based checklist*	To tailor information might help the patient to prepare for their first visit	1495 consecutive patient visits	+++	internal medicine clinic	USA	General	Pre-assessment tools	Demographic data, Medical history, Reason for referral, Symptoms, Medication	Patient-provider communication, Visit length	14
38	Wolff, J. L. et al. [46]	2014	J Am Geriatr Soc	*Paper-based checklist*	To explore whether a pre-consultation web-based intervention enables patients with diabetes to articulate their agenda in a consultation	Thirty-two patients age 65+	+++	Geriatric clinic	USA	Older patients	Pre-assessment tools	Demographic data, Medical history, Patient awareness, Symptoms, Medication	Perceived involvement in care, Patient expectations in consultations	17
39	Causarano, N. et al. [41]	2015	Support Care Cancer	*Online and offline sources of information and support*	To compare the acceptability and feasibility of using brief electronic versus paper screening	41 patients	+++	plastic surgery clinic	Canada	Breast Cancer	Patient education	Demographic data, Patient awareness	Patient expectations in consultations, Medication, and treatment adherence	15
40	Grant, R. W. et al. [44]	2008	Arch Intern Med	*EHR-linked care program*	To evaluate a patient chart information in preparation for a scheduled office visit	244 patients with DM	+++	primary care	USA	Diabetes	Decision aid tools, Pre-assessment tools, Patient education	Demographic data, Medical history, Lifestyle, Family history, Lab data, Reason for referral, Patient awareness, Drug side effects Symptoms, Medication	Patient-provider communication, Illness perceptions, Medication and treatment adherence, Symptom control	12
41	Brackett, C., & Kearing, S [62].	2015	Patient	*Online and offline sources of information and support*	To determine whether a brief pre-visit questionnaire can improve primary care provider	11,493 patients	+++	Cancer clinic	USA	Cancer	Patient education	Demographic data, Patient awareness	Patient expectations in consultations, Mental health topics, Visit length	15
42	Meropol, N. J. et al. [33]	2013	Cancer	*Electronic pre-office visit checklist*	To assess the acceptability of a pre-consultation checklist for older patients	1932 patients	+++	Cancer clinic	USA	Cancer	Decision aid tools, Pre-assessment tools	Demographic data, Medical history, Family history, Lab data, Reason for referral, Drug side effects Symptoms, Medication	Illness perceptions, Perceived involvement in care, Patient expectations in consultations, Visit length	15
43	Kim-Hwang, J. E. et al. [49]	2010	J Gen Intern Med	*EHR-linked care program*	Bridging the gap about post-mastectomy breast by applying a new approach	540 questionnaires	+++	Primary care	USA	General	Decision aid tools, Pre-assessment tools, Patient education, Reminders	Demographic data, Medical history, Family history, Reason for referral, Symptoms, Medication	Patient-provider communication, Illness perceptions, Perceived involvement in care, Patient satisfaction, Medication and treatment adherence, Adherence to visit scheduling	16
44	Muraywid, B. et al. [63]	2020	J Manag Care Spec Pharm	*EHR-linked care program*	To evaluate the impact of a DMSPECIFIC PHR	700 patients	+++	Primary care	Colombia	Diabetes	Decision aid tools, Pre-assessment tools, Patient education, Reminders	Demographic data, Medical history, Reason for referral, Symptoms, Medication	Patient-provider communication, Illness perceptions, Patient satisfaction, Appointment intake information, Adherence to visit scheduling, Quality of life	9
45	Vo, M. T. et al. [34]	2019	Journal of General Internal Medicine	*Electronic pre-office visit checklist*	To facilitate shared decision-making by utilizing a web-based survey system before the visit.	1276 patients	+	primary care	USA	Diabetes	Decision aid tools, Pre-assessment tools	Demographic data, Medical history, Lab data, Reason for referral, Symptoms, Medication	Patient-provider communication, Perceived involvement in care, Patient satisfaction	19
46	Baker, D. W. et al. [73]	2011	Journal of the American Medical Informatics Association	*EHR-linked care program*	To develop an intervention to improve communication between patients and their physicians	12,288 patients	+++	Internal medicine	USA	General	Decision aid tools, Pre-assessment tools	Demographic data, Medical history, Lab data, Reason for referral, Symptoms, Medication	Patient-provider communication, Perceived involvement in care, Patient satisfaction, Adherence to visit scheduling, Self-care, Symptom control	16
47	Grant, Richard W, et al. [35]	2019	The Annals of Family Medicine	*Electronic pre-office visit checklist*	To improve patient values and needs.	750 English- or Spanish-speaking patients	+++	Primary care	Canada	General	Decision aid tools, Pre-assessment tools	Demographic data, Medical history, Lab data, Reason for referral, Symptoms, Medication	Patient-provider communication, Patient satisfaction, Appointment intake information	17
48	Harrington, J. T., & Walsh, M. B [67]	2001	Arthritis Care & Research: Official Journal	*EHR-linked care program*	To determine the impact of e-referral and pre-visit planning.	270 patients	+++	Rheumatology	USA	Rheumatology diseases	Decision aid tools, Pre-assessment tools, Patient education, Reminders	Demographic data, Medical history, Family history, Reason for referral, Symptoms, Medication	Patient-provider communication, Illness perceptions, Perceived involvement in care, Patient satisfaction, Medication and treatment adherence, Adherence to visit scheduling	10
49	Gadomski, A. M et al. [71]	2015	Journal of Adolescent Health	*EHR-linked care program*	Their objective was to improve health outcomes and reducing costs.	72 patients	+++	pediatric primary care	USA	Mental health	Decision aid tools, Pre-assessment tools, Patient education, Reminders	Demographic data, Medical history, Family history, Reason for referral, Symptoms, Medication	Patient-provider communication, Perceived involvement in care, Patient satisfaction, Appointment intake information, Medication adherence, Adherence to visit scheduling, Reductions in prescription costs, Mental health topics	14

**Fig. 3:**
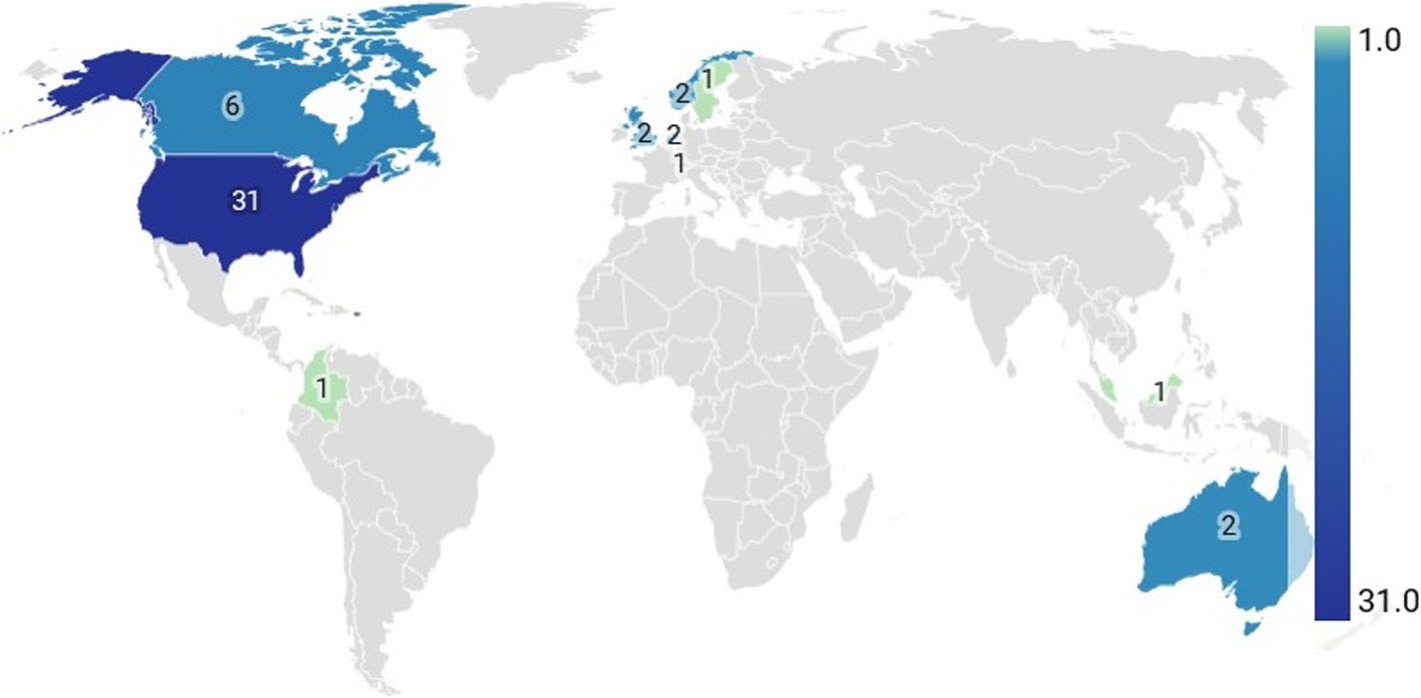
The distribution of studies based on their conducted countries worldwide

Analysis of studies showed that the application of pre-visit planning is the most favorite of developed countries. Of them, the USA has the most contribution among other studies. After that Canada ranks second in the deployment plan is allocated to pre-visit intervention. The distribution of studies concerning the country is shown in Fig. 3.

### Different techniques for putting pre-visit into practice

The investigation showed that pre-visit can apply in different ways regarding timing, main approaches, and types. The analysis showed that different types of pre-visit techniques have been employed by authors to facilitate office visits and patient care. All of these plans can be categorized into eight different categories, utilizing an electronic pre-office checklist with 12 studies (24.5%) [25, 29–35, 48, 58, 59, 68], educating patients and support them before each visit in form of online and offline source of information with 12 studies (24.5%) [23, 36–42, 60–62, 66, 74], applying an EHR-linked care program with different checklists and assessment tools with nine studies (18.4%) [22, 43, 44, 49, 63, 67, 70, 71, 73], using paper-based checklists with nine studies (18.4%) [45, 46, 50–52, 64, 65, 69, 72], preparing and assess patient with the pre-visit phone-based intervention with two studies (4.1%) [24, 53], using self-triage or self-assessment tools with two studies (4.1%) [54, 55], using automatic reminders and sheets with one article (2%) [56], and using pre-clinic consultation by other health care team member with one article (2%) to prepare the patient for each visit [57].

According to findings, the most favorite types of pre-visit model were related to using electronic pre-office visit checklists and supporting patients by providing them with the necessary information in the form of online and offline training. In three articles, this information was provided to patients in the form of educational websites [37, 39, 40, 60], while in the other six articles, the information was provided to patients in the form of training sessions before the patient’s visit and referring to the clinic [23, 36, 38, 41, 42, 61, 62, 66].

The next widely applied method was the EHR-linked care program that put pre-visit planning into practice. Ten articles used pre-visit solutions such as electronic checklists, automated reminders, decision-making tools, and reviewable forms that could be implemented by connecting to electronic medical records. In third place, there are paper-based checklists used for patient preparation with nine papers. These checklists included questions about demographic information, the main problems, medical history, general symptoms, illness history, hospitalizations, medications, family history of a specific illness, level of education, location, and questions about the patient’s lifestyle. Other solutions were used in a smaller number of articles. Regarding pre-visit counseling, only one article applied the consultation of clinical pharmacists before the office visit. This approach leads to providing the physician with better information after the initial completion of the medical record.

In terms of timing, pre-visit intervention could be conducted at a different time in the patient care process. Taken together, all of these possibilities could be categorized into four situations. It can be occurred before each visit, between visits, at the end of each visit on the current visit, and in a combination of the previous three models. Our results showed that almost 81% of studies were related to procedures that were done between each visit, while 42% of articles were related to procedures that were done before each visit. Only 10 % of studies were conducted at end of the current visit.

In terms of main approaches, the analysis of studies showed that all studies can be divided into three main categories based on the main approaches. These three approaches comprise, improving the current visit and preparing the patient for the next visit, perform some procedures for patient preparedness such as sending reminders or filling pre-visit checklists, and providing more inclusively insight about the patient for the physician before they come in for an office visit. The final analysis of the studies based on the main objectives and the timing is summarized in Table 3. Out of 49 studies, the main approach forty-eight of articles were related to patient preparedness and enhance patient adherence to their treatment.

**Table 3:** Results of study analysis based on main objectives and timing

Author	*Pre-Visit Model*	Main Approaches			Timing		
		Improve the current visit	Patient preparedness	providing inclusive insight for physician	Before each visit	Between each visit	At end of the current visit
Allende-Richter, S. et al.	*Paper-based checklist*		√				
Rivo, J. et al.	*Phone-based pre-office visit preparation*		√			√	
Cox, N et al.	*A pre-clinic care team consultation*			√		√	
Page.T et al.	*Phone-based pre-office visit preparation*		√			√	
Bose-Brill, S et al.	*EHR-linked care program*	√	√	√	√		
Riese, A et al.	*Electronic pre-office visit checklist*		√			√	
Myers, P et al.	*Online and offline sources of information and support*	√	√	√	√		
Frank, O et al.	*Paper-based checklist*		√	√	√		
Lewin, W et al.	*Paper-based checklist*		√			√	
Liu, T et al.	*Electronic pre-office visit checklist*		√			√	
Stankowsk, T. J et al.	*Online and offline sources of information and support*		√			√	
Wald, J. S et al.	*EHR-linked care program*		√			√	
Zanini, C et al.	*Paper-based checklist*		√		√	√	
Grant. R et al.	*EHR-linked care program*	√	√	√			
Frank, O. R et al.	*Paper-based checklist*		√			√	
Rodenbach, R. et al.	*Online and offline sources of information and support*		√			√	
Hitchings, S., and Barter, J.	*Self-triage or self-assessment tool*		√			√	
Sleath, B et al.	*Online and offline sources of information and support*	√	√		√		√
Tucholka, J. et al.	*Online and offline sources of information and support*		√			√	
Aboumatar, H et al.	*Online and offline sources of information and support*		√			√	
Albada, A. et al. (2012)	*Electronic pre-office visit checklist*		√	√		√	
Bruce, J. G. et al.	*Online and offline sources of information and support*		√			√	
Walker, M. E. et al.	*Paper-based checklist*		√			√	
Savage, C. et al.	*Paper-based checklist*		√		√	√	
Judson, T. J. et al.	*Self-triage or self-assessment tool*		√	√	√	√	
Albada, A. et al. (2015)	*Electronic pre-office visit checklist*		√			√	
Purkaple, B et al.	*Paper-based checklist*		√		√	√	
Krist, A. H. et al.	*Online and offline sources of information and support*		√	√	√	√	
Fothergill, K. et al.	*Electronic pre-office visit checklist*	√	√	√	√		
Lee, Y. K et al.	*Electronic pre-office visit checklist*	√	√		√		√
Johansen, M. et al.	*Electronic pre-office visit checklists*		√			√	
Hu, X et al.	*Online and offline sources of information and support*		√		√	√	
Albada, A. et al.	*Online and offline sources of information and support*		√	√	√	√	
Frost, J. et al.	*Electronic pre-office visit checklist*		√			√	
O’Brien, M et al.	*Electronic pre-office visit checklist*		√	√		√	
Wald, J. S. et al.	*EHR-linked pre-visit checklist*	√	√	√	√		
Albertson, G. et al.	*Paper-based checklist*		√			√	
Wolff, J. L. et al.	*Paper-based checklist*		√			√	
Causarano, N. et al.	*Online and offline sources of information and support*		√			√	
Grant, R. W. et al.	*EHR-linked care program*		√	√	√	√	
Brackett, C, & Kearing, S.	*Online and offline sources of information and support*		√	√	√	√	
Meropol, N. J. et al.	*Electronic pre-office visit checklist*		√	√	√	√	
Kim-Hwang, J. E. et al.	*EHR-linked care program*		√	√	√	√	
Muraywid, B. et al.	*EHR-linked care program*	√	√	√	√	√	√
Vo, M. T. et al.	*Electronic pre-office visit checklist*		√			√	
Baker, D. W. et al.	*EHR-linked care program*		√			√	
Grant, R et al.	*EHR-linked care program*		√			√	
Harrington, J, & Walsh, M	*EHR-linked care program*	√	√	√	√	√	√
Gadomski, A. M et al.	*EHR-linked care program*	√	√	√	√	√	√
***Total***		**10**	**48**	**18**	**21**	**40**	**5**

**Fig. 4:**
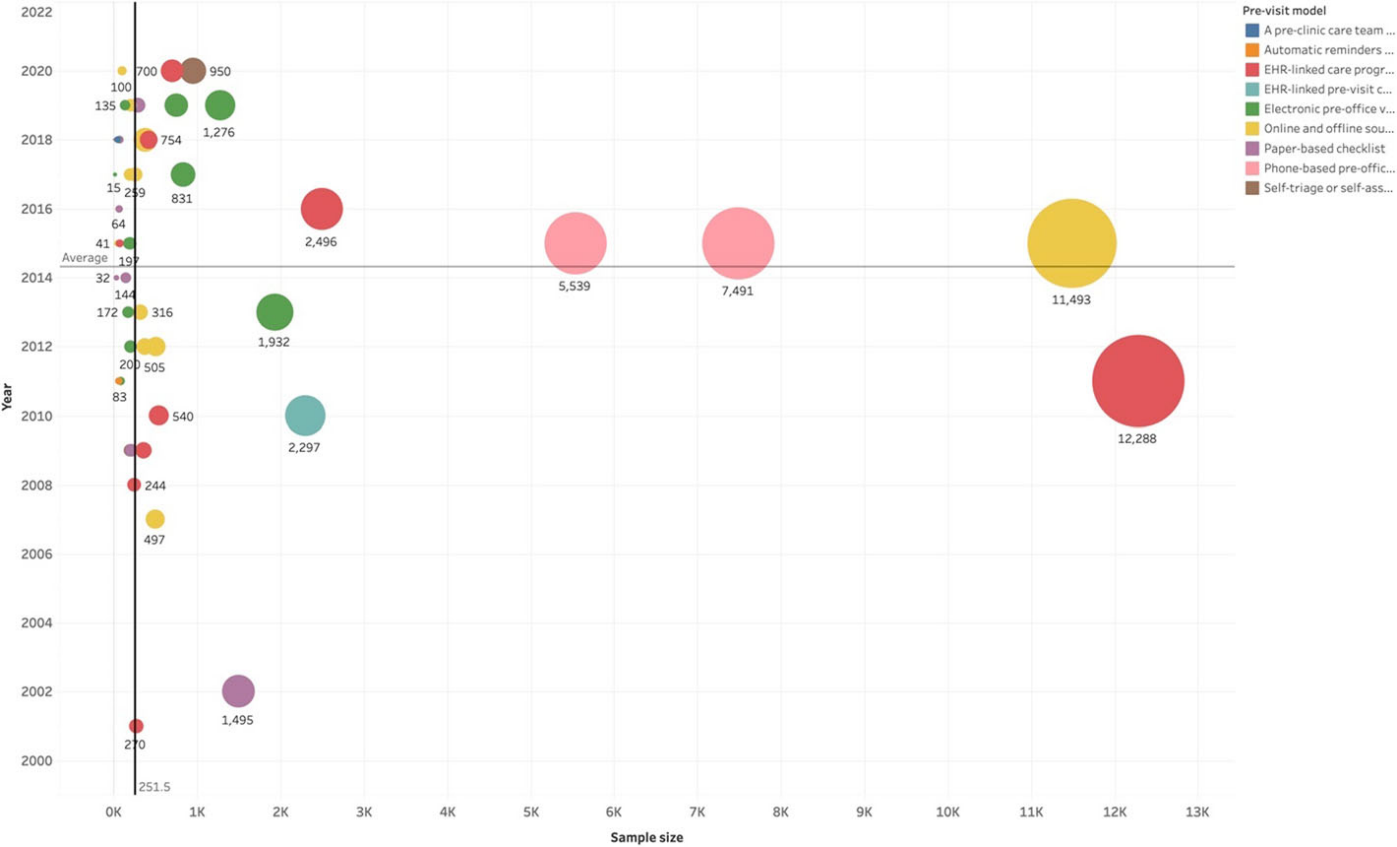
Distribution of studies based on sample size, year, and different techniques

Out of 49 studies, only one study did not report the sample size of their study. In total, the sample size ranged from 15 to 12,228 with a mean sample size of 1160.3877 (SD = ± 2613.799). In Fig. 4, the distribution of studies based on sample size, year, and different techniques are represented.

### The effectiveness of pre-visit planning

**Fig. 5:**
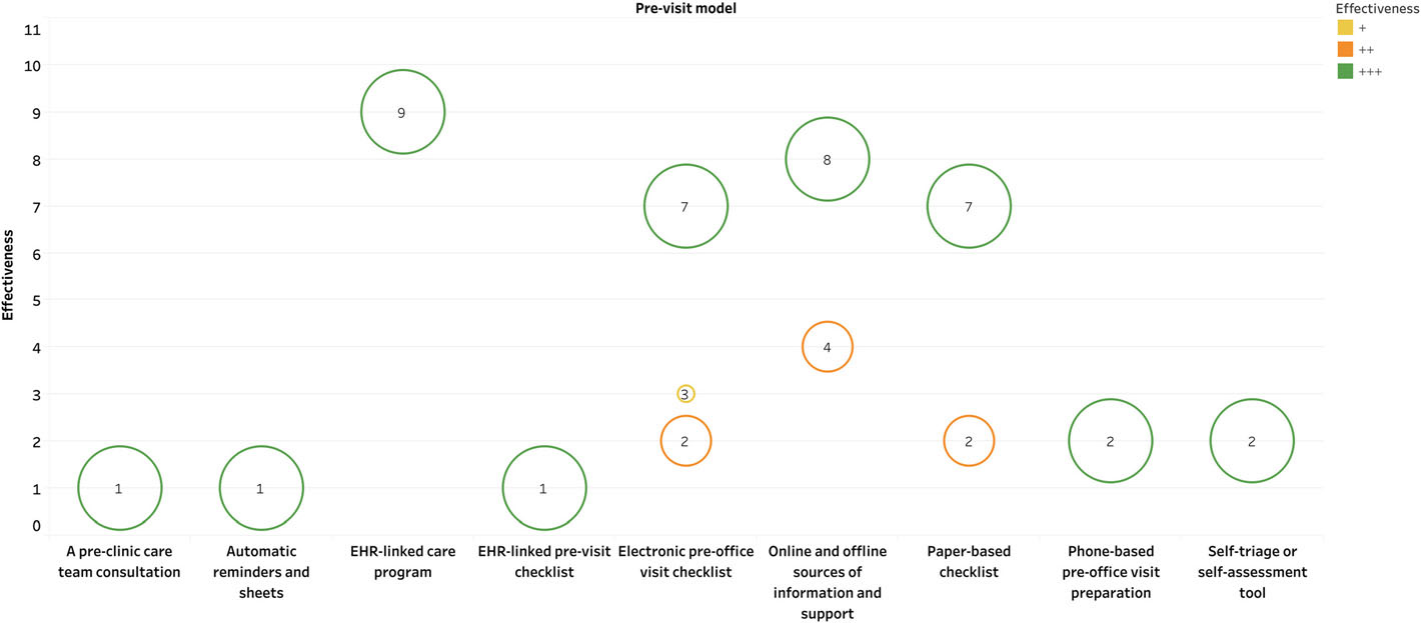
Effectiveness of studies concerning applied methods

Articles were also reviewed regarding the effectiveness of the applied methods. Out of 49 studies, the authors of 41 articles (83.67%) considered pre-visit planning to be effective in clinical practice. While six studies (12.24%) reported the effectiveness of these tools as moderate, only two articles (4.08%) believed that using this method had very little effect on improving patient-centered care. The effectiveness of studies concerning applied methods is shown in Fig. 5.

The effectiveness has been reported by researchers using various outcome measures in studies. These outcome measures reported in reviewed articles, along with their frequency and their effectiveness, are shown in Table 4.

**Table 4:** Outcome measures reported in these articles with their frequency and their effectiveness

Outcome measure	Low	Medium	High	Total
Patient-provider communication	1	4	21	26
Illness perceptions and knowledge	1	5	15	21
Perceived involvement in care	3	3	14	20
Patient satisfaction	1	5	12	18
Patient expectations in consultations		5	12	17
Appointment intake information	2	4	11	17
Medication and treatment adherence	1	2	9	12
Adherence to visit scheduling		2	9	11
Identifying referral appropriateness		2	9	11
Visit length		2	7	9
Symptom control		1	5	6
Mental health topics		2	4	6
Self-care	2		3	5
Intention-to-treat (ITT) analysis			2	2
Quality of life			2	2
Patient waiting times		2		2
Reductions in prescription costs			1	1

### Different diseases and the main reason for referral

**Fig. 6:**
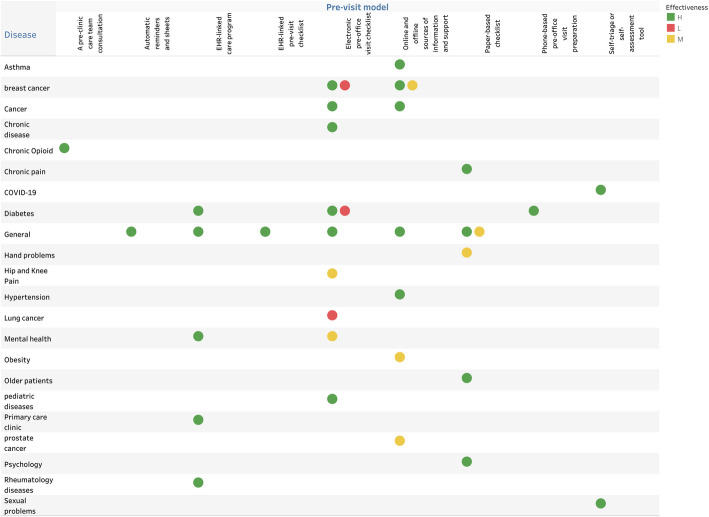
Frequency of disease regarding applied methods and their effectiveness

Through this survey, the referred clinic and the main reasons for the referral were also examined in reviewed articles. In terms of the reason for referral and diseases, the most common reason for referral was related to chronic disease and general problems. The frequency of disease regarding applied methods and their effectiveness are represented in Fig. 6. Regarding the type of clinic that was considered for implementing pre-visit planning, the highest frequency was related to primary care clinics. Next, surgical clinics had the largest number of pre-visit programs.

### Information and collected data

**Fig. 7:**
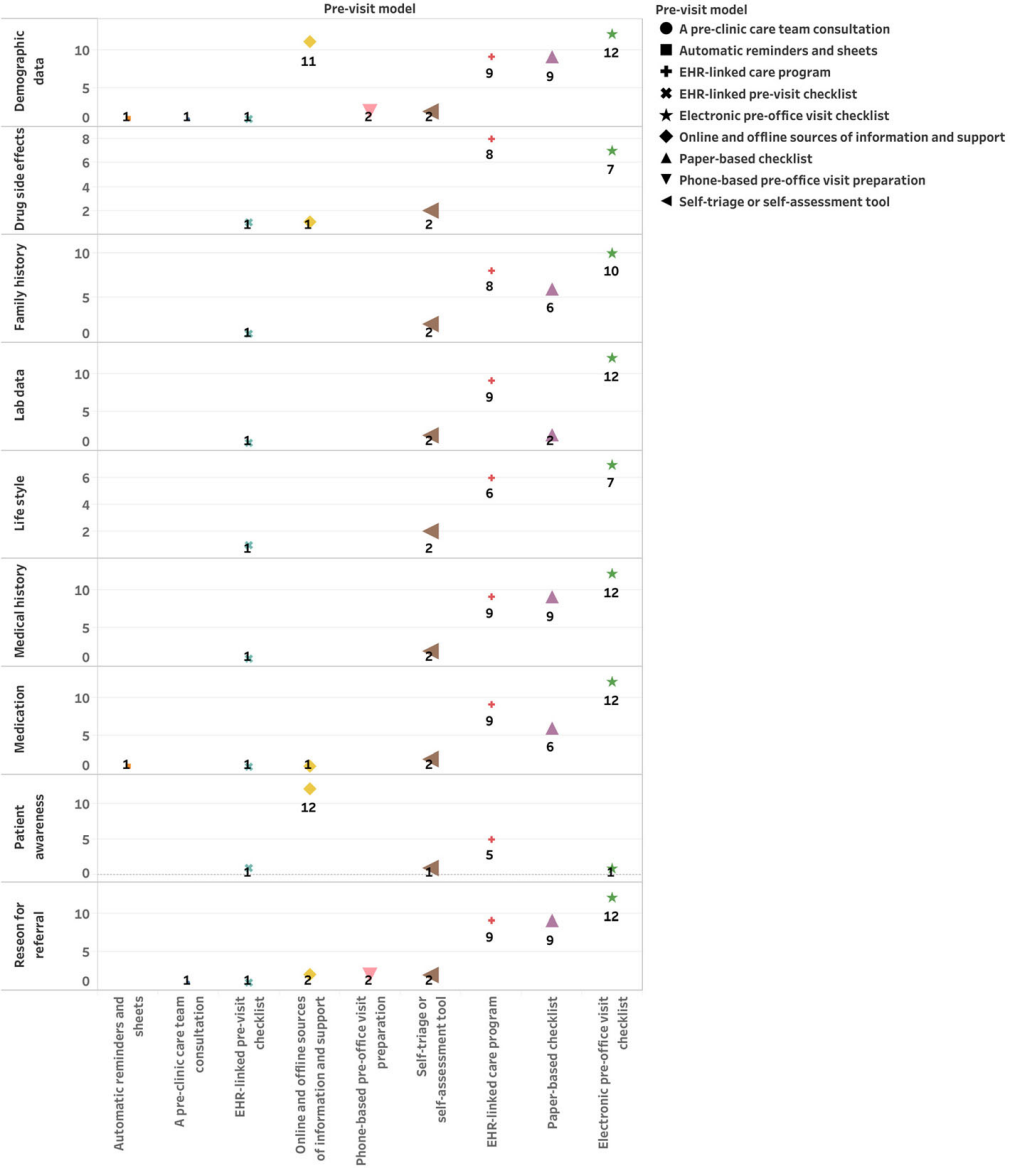
Distribution of different kinds of collected data regarding pre-visit techniques

To implement pre-visit planning, various types of data and information have been collected in studies. These collected data were very diverse. Hence, these different types of information can be divided into nine categories concerning their application. The different types of information regarding applied techniques are shown in Fig. 7.

### Determining the main categories of applied techniques regarding medical informatics

**Fig. 8:**
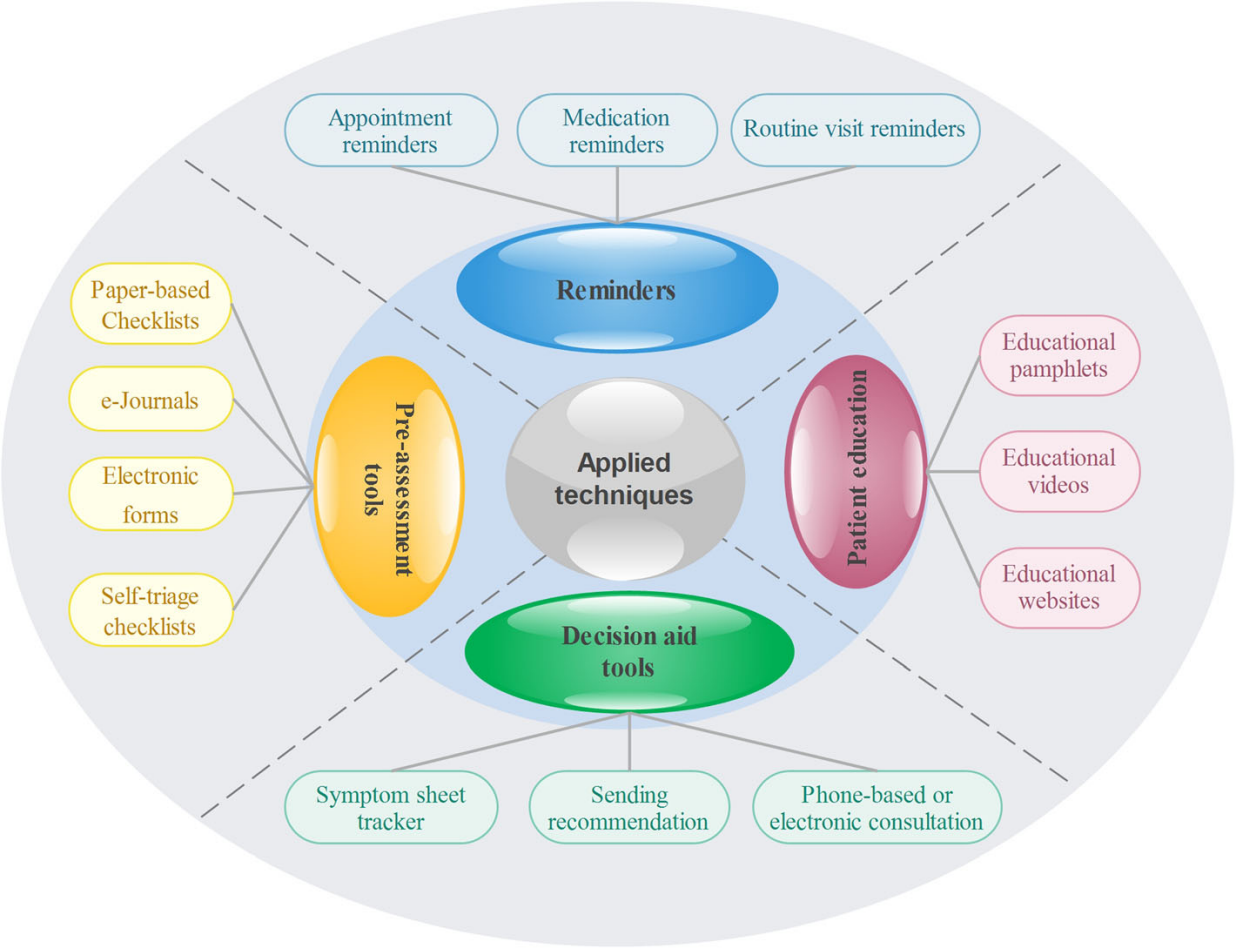
The main applied techniques through pre-visit planning in terms of medical informatics

Coding of all research studies and extracted themes using thematic analysis leads to discover the main sub-themes in terms of medical informatics. Therefore, all of the employed techniques can be divided into four categories, pre-assessment forms, educational resources, decision aid tools, and reminders as the main themes. The main themes and sub-themed are shown in Fig. 8. Different aspects of such a model were shaped by mapping the main concepts obtained through this survey. The details of applied techniques in terms of the medical informatics view are described in Table 2.

### Framework suggestion and IT-based solution

**Fig. 9:**
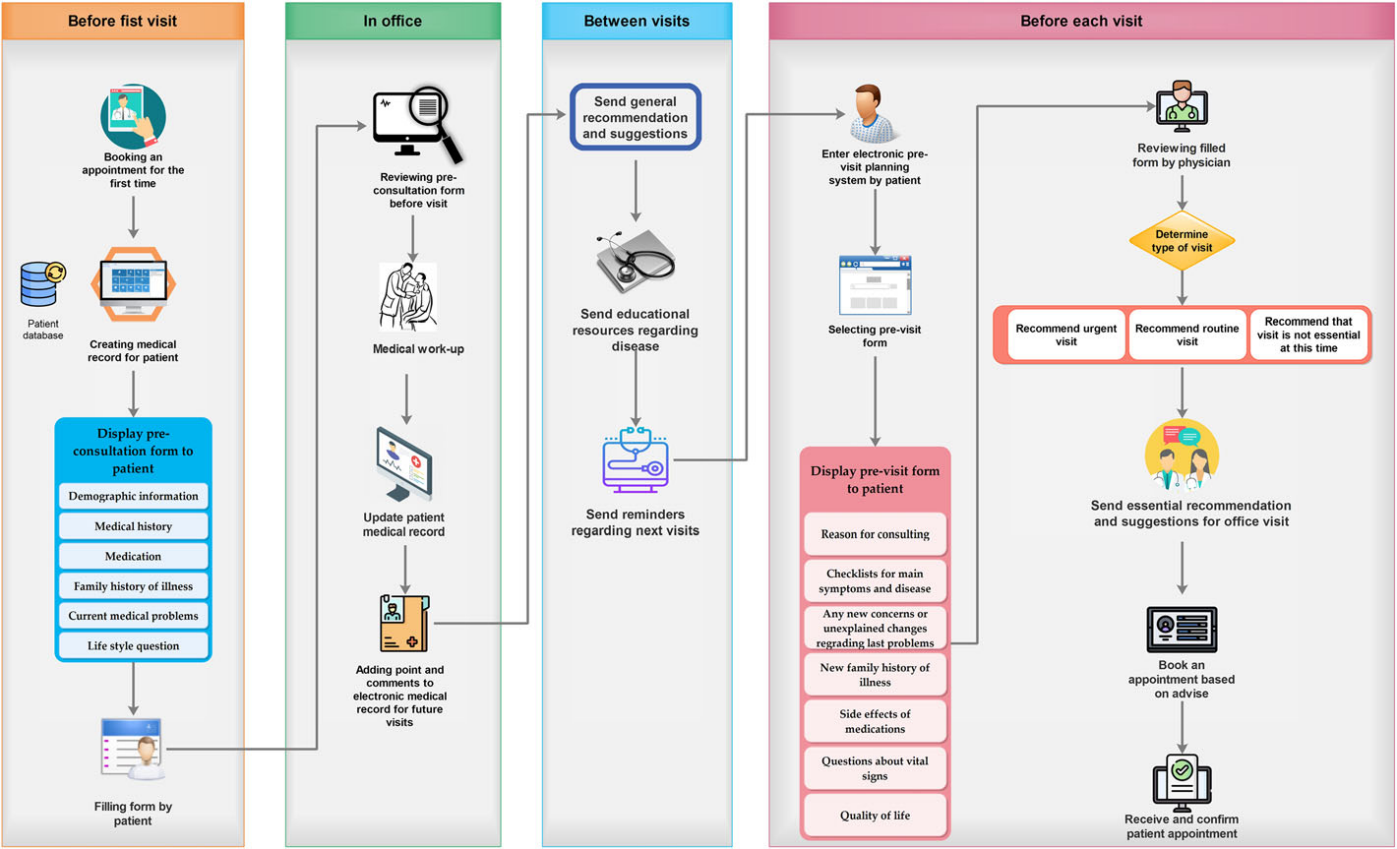
The overall model of pre-visit planning care

After a qualitative analysis of the results based on predetermined categories, the main ideas can be summarized in a proposed framework as an electronic-based advanced care program. Based on the results, this model is divided into four main parts in terms of time. This model is represented in Fig. 9. In this model, the main focus is on the patient. The workflow is designed to improve the relationship between physician and patient in the simplest way. It is done by involving the patient in their care, which is one of the main purposes of using pre-visits in studies.

In this model, it is assumed that an electronic system is available to manage patient information. To implement a pre-visit-based program, a section is also considered for patient access to his care plan in the proposed model. Based on this model, the patient can pursue the main goals of pre-visit planning through suggested workflow, such as disease management, treatment adherence, receiving the necessary advice and training, and preparing for each visit. To increase the effectiveness of the devised model, it is suggested that the proposed system should have interacted with existing databases and electronic health systems.

## Discussion

### Summary of findings

This survey summarized the characteristics of published studies on pre-visit planning and its application in various health domains. To our knowledge, this study represents the first overview of the existing evidence about the different pre-visit planning techniques in clinical practice. Forty-nine articles from ten countries were included in this survey. As mentioned in the results, these techniques can classify into eight categories. Among them, the most widely used methods are related to using electronic pre-office visit checklists and supporting patients by providing them with the necessary information in the form of online and offline educational resources.

Consistent with the present findings, our results showed that applying pre-visit techniques was not restricted to office visits [75]. So, pre-consultation planning can employ before each patient’s consultation, between the patient’s visits, and during the current visit to facilitate complicated patient care process.

One of the remarkable results of this study is that this approach has been used more in developed countries. It may be because it is easier to take a participatory approach to patient care in developed countries due to a high level of patient literacy.

### Results in the context of other researches

Our results showed that most studies have been conducted with the main goal of preparing the patient by involving them in their treatment process. Patient preparedness had the most impact on the patient’s perceptions of his disease and overall patient satisfaction [76]. Similarly, Ringdal et al. [77] indicated through their survey that patients were satisfied with their active role as a partner on the healthcare team. Also, this is exactly in line with the main goal of the patient-centered care paradigm regarding the individualized approach to the patient’s treatment [78–80].

However, Geraghty et al. [81] showed through their study that there is a linear relationship between patient satisfaction and visit length. Unfortunately, long waits are common at outpatient clinics [82]. Hence, our results illustrated that using a pre-visit assessment tool such as a simple checklist or questionnaire is almost effective to maximize the available time during a consultation for making the best decisions by physicians. Also, it can provide better insight for physicians to better communicate with the patient by knowing the patient’s background during the consultation [22, 33, 35, 47, 54, 60, 64, 67, 69, 70, 83–88].

Analysis of results revealed that most studies considered the pre-visit assessment tool as an independent solution that was not connected to existing electronic systems. However, in some studies, a comprehensive care plan has been taken. A pre-visit planning program could be linked to a patient’s electronic medical record as used in some reviewed studies. This approach is similar to the motivational interviewing (MI) technique that is applied to improve patient-centeredness in other studies. Motivational interviewing is a technique to help patients address their chief problems and increase their understanding of their participatory role in the treatment process [89].

### Implications for research and practice

Planned and targeted care is one of the main components of the patient-centered care model [79]. Hence, implementing pre-visit tools within an advanced planned care program might be more effective in moving towards effective patient-centered care. However, pre-visit planning care is a new approach, no framework or conceptual model was introduced according to this subject. Only a planned care model was introduced by the Health Research and Quality Agency as a comprehensive patient-centered medical home (PCMH) approach in which one of its main components is pre-visit planning [90, 91]. Hence, our findings are summarized in a conceptual model regarding applying the pre-visit assessment tool in electronic-based planned patient care (Fig. 9). However, the EHR-linked pre-visit type was used only in the nine studies, the suggested model is not devised in a stand-alone model. Nowadays, with the advent of the digital age, applying integrative electronic systems and medical informatics-based solutions are inevitable [92].

One of the significant gaps that were mentioned in the studies is the unnecessary referrals of patients to outpatient offices [93, 94]. These unnecessary visits in the event of pandemics can also lead to the spread of disease [95, 96]. In such a framework, avoiding unnecessary referrals was considered to fight the pandemic. Such an approach can be useful to prevent the spread of the COVID-19 disease too.

### Limitations

Since this study is the first attempt to review and analyze the published articles regarding pre-visit planning, it encounters some limitations. The results of some studies might be published in the form of reports, letters to the editor, or other types of study. Thus, we might not have considered them based on our exclusion criteria. The results showed that most studies point out pre-visit planning conducted by large institutions and reputable organizations; their data are absurdly confounded by the fact that better-funded institutions probably produce better outcomes. Also, some researchers might put pre-visit into practice but they did not publish their attempts in form of any research article or conference paper. It could cause publication bias. Thus, further researches for specific domains in clinical practices might be done in the future.

## Conclusion

Using a systematic review approach leads to get a comprehensive overview of literature conducted in the use of various pre-visit approaches. Our results revealed that the direct outcome of planning a pre-visit care program was enhancing the quality of patient care alongside enhancement patient-provider communication. Improving the patient-physician relationship is a key factor in moving towards a patient-centered care paradigm. The qualitative and thematic analysis of the articles also showed that pre-visit planning has the greatest impact on the relationship between physician and patient. It can account for such a useful tool to move toward patient-centered care. However, such an approach can also be helpful to control pandemic diseases by reducing unnecessary referrals. Thus, the application of pre-visit tools can be considered as one of the key components of designing a patient-centered care system. In this survey, we tried to summarize our findings and our suggestions in a complete patient care framework based on pre-visit planning techniques.

## Supplementary Information

**Additional file 1: Table A-1.** Applied search strategies and their results.

## Data Availability

The study involves only a review of the literature without involving any data.

## Abbreviations

PRISMA: Preferred Reporting Items for Systematic Reviews and Meta-Analyses
PCC: Patient-centered care
