# Supplementary material for: Applied techniques for putting pre-visit planning in clinical practice to empower patient-centered care in the pandemic era: a systematic review and framework suggestion
Source: BMC Health Serv Res. 2021 May 13;21:458. doi: 10.1186/s12913-021-06456-7 (PMC8116646; doi:10.1186/s12913-021-06456-7)
Supplement: Supplementary file 1 — Additional file 1: Table A-1. Applied search strategies and their results. [file 12913_2021_6456_MOESM1_ESM.docx]

**Appendix:**

| **Database** | **Search strategy** | **Results** |
| --- | --- | --- |
| **PubMed** | (("self-triage"[Title/Abstract]) OR ("selftriage"[Title/Abstract]) OR ("pre-consultation"[Title/Abstract]) OR ("pre-visit"[Title/Abstract]) OR ("previsit"[Title/Abstract]) OR ("previsit assessment"[Title/Abstract]) OR ("preconsultation"[Title/Abstract]) OR ("pre-appointment evaluation"[Title/Abstract]) OR ("pre-visit planning"[Title/Abstract]) OR ("previsit planning"[Title/Abstract]) OR ("pre-appointment assessment"[Title/Abstract]) OR ("pre-visit screening"[Title/Abstract]) OR ("previsit screening"[Title/Abstract]) OR ("previsit computerized screening"[Title/Abstract])) AND (("Delivery of Health Care"[Mesh]) OR ("Delivery of Health Care"[Title/Abstract]) OR ("Health Care Delivery"[Title/Abstract]) OR ("Health Care Systems"[Title/Abstract]) OR ("Health Care System"[Title/Abstract]) OR ("Physician-Patient Relations"[Mesh]) OR ("Advance Care Planning"[MeSH Terms]) OR ("Advance Health Care Planning"[Title/Abstract]) OR ("Advance Medical Planning"[Title/Abstract]) OR ("Patient Care Planning"[Mesh]) OR ("Patient-Centered Care"[Mesh]) OR ("Patient Centered Care"[Title/Abstract])) | 307 |
| **Web of Science** | (TS=("Patient Care Planning") OR TS=("Advance Medical Planning") OR TS=("Advance Health Care Planning") OR TS=("Delivery of Health Care") OR TS=("Delivery of Care") OR TS=("Health Care Delivery") OR TS=("Health Care Systems") OR TS=("Health Care System") OR TS=("Healthcare Delivery") OR TS=("healthcare planning") OR TS=("Physician-Patient Relations") OR TS=("health care planning")) AND (TS=(("pre-visit")) OR TS=("previsit computerized screening") OR TS=("previsit screening") OR TS=("previsit consultation") OR TS=("pre-visit screening") OR TS=("pre-visit planning") OR TS=("previsit planning") OR TS=("pre-appointment evaluation") OR TS=("previsit assessment") OR TS=("self triage") OR TS=("self-triage") OR TS=("pre visit") OR TS=("Patient-Centered Care")) | 9 |
| **Scopus** | ( TITLE-ABS-KEY ( "pre-visit" )  OR  TITLE-ABS-KEY ( "previsit" )  OR  TITLE-ABS-KEY ( "previsit consultation" )  OR  TITLE-ABS-KEY ( "self-triage" )  OR  TITLE-ABS-KEY ( "self triage" )  OR  TITLE-ABS-KEY ( "previsit assessment" )  OR  TITLE-ABS-KEY ( "pre-appointment evaluation" )  OR  TITLE-ABS-KEY ( "previsit planning" )  OR  TITLE-ABS-KEY ( "pre-visit planning" )  OR  TITLE-ABS-KEY ( "pre-visit screening" )  OR  TITLE-ABS-KEY ( "previsit screening" )  OR  TITLE-ABS-KEY ( "previsit computerized screening" ) )  AND  ( TITLE-ABS-KEY ( "Delivery of Health Care" )  OR  TITLE-ABS-KEY ( "Delivery of Care" )  OR  TITLE-ABS-KEY ( "Health Care Delivery" )  OR  TITLE-ABS-KEY ( "Health Care Systems" )  OR  TITLE-ABS-KEY ( "Health Care System" )  OR  TITLE-ABS-KEY ( "Advance Care Planning" )  OR  TITLE-ABS-KEY ( "Physician-Patient Relations" )  OR  TITLE-ABS-KEY ( "Advance Care Planning" )  OR  TITLE-ABS-KEY ( "Advance Health Care Planning" )  OR  TITLE-ABS-KEY ( "Advance Medical Planning" )  OR  TITLE-ABS-KEY ( "Patient Care Planning" ) OR TITLE-ABS-KEY ( "Patient-Centered Care" ) ) | 69 |

Table A-1- Applied search strategies and their results
